# Treatment effects of vitamin D3 and marine omega‐3 fatty acids on plasma ADRD biomarkers: Exploring sex and race differences

**DOI:** 10.1002/alz.090064

**Published:** 2025-01-09

**Authors:** Chirag M. Vyas, Beth L. Ostaszewski, Amirah K. Anderson, Nancy R. Cook, Jae H. Kang, Jennifer R. Gatchel, David Mischoulon, Grace Chang, Charles F. Reynolds, JoAnn E. Manson, Dennis J. Selkoe, Olivia I. Okereke

**Affiliations:** ^1^ Department of Psychiatry, Massachusetts General Hospital, Harvard Medical School, Boston, MA USA; ^2^ Department of Neurology, Brigham and Women's Hospital, Harvard Medical School, Boston, MA USA; ^3^ Department of Medicine, Brigham and Women's Hospital, Harvard Medical School, Boston, MA USA; ^4^ Channing Division of Network Medicine, Brigham and Women’s Hospital, Harvard Medical School, Boston, MA USA; ^5^ VA Boston Healthcare System, Harvard Medical School, Boston, MA USA; ^6^ University of Pittsburgh Medical Center, Pittsburgh, PA USA

## Abstract

**Background:**

There is an urgent need to identify novel, accessible and affordable strategies to prevent cognitive decline and progression in the Alzheimer disease and related dementias (ADRD) continuum. Vitamin D3 and marine omega‐3 fatty acids (omega‐3s) supplements show promise for cognitive protection, with potential variations in their effects by sex or race. However, to date, no randomized clinical trials (RCTs) have tested their impact on emerging plasma‐based biomarkers with potential utility to predict ADRD pathogenesis.

**Methods:**

Vitamin D and Omega‐3 Trial (VITAL) is a completed, nation‐wide, 2x2 factorial placebo‐controlled RCT testing vitamin D3 (2000 IU/d) and omega‐3s (1 g/d) for cancer and cardiovascular disease prevention. We included a Boston‐area sub‐cohort of 929 randomized VITAL participants who provided blood samples at baseline, 2‐year, and/or 4‐year follow‐up. Plasma ADRD biomarkers, including an N‐terminal tau fragment (NT1), amyloid‐β (Aβ)‐40, Aβ‐42, neurofilament‐light (NfL), and glial fibrillary acidic protein (GFAP), were measured. We used multivariable‐adjusted repeated measures models for data analysis; sex and race were pre‐specified effect modifiers.

**Results:**

Among 929 participants, the mean age was 65 years; 49.2% were females; 17.3% were from racial and/or ethnic minority backgrounds, including 8.2% Black adults. Neither vitamin D3 nor omega‐3s, compared to placebo, significantly reduced ADRD biomarkers overall across 4 years of treatment; however, there was a trend for reduction in Aβ‐40:Aβ‐42 ratio over 4 years for vitamin D3 versus placebo [percent difference (95% confidence interval [CI]): ‐1.2 (‐2.7, 0.3)]. Subgroup analyses uncorrected for multiple‐testing suggested interactions by sex and race. Vitamin D3 versus placebo resulted in a reduction in NT1 among males (‐2.7%) but not females (p‐interaction=0.08). Among Black participants, vitamin D3 versus placebo resulted in a 16% reduction in NfL levels [95% CI, ‐30.5% to 1.1%; p‐interaction=0.06], while omega‐3s versus placebo showed a 12.4% reduction in GFAP levels [95% CI, ‐21.5% to ‐2.2%; p‐interaction=0.049].

**Conclusion:**

In this RCT sub‐cohort of 929 older adults, neither vitamin D3 nor omega‐3s supplements significantly reduced selected plasma ADRD biomarkers over 4 years. We observed potential differences by sex and race in reductions of some ADRD biomarkers in response to these supplements which warrant further investigation in a larger sample.

